# High-Mileage Running-Induced Heel Strike Anemia in a 46-Year-Old Female

**DOI:** 10.7759/cureus.115742

**Published:** 2026-09-03

**Authors:** Mariam Aamir, Zachary Fitzgerald, Collin Kitchell

**Affiliations:** 1 Medical School, Edward Via College of Osteopathic Medicine, Spartanburg, USA; 2 Family Medicine, Cleveland Clinic, Vero Beach, USA

**Keywords:** anemia, distance running, foot strike hemolysis, heel strike anemia, march hemoglobinuria, runner

## Abstract

Foot-strike hemolysis is an underrecognized cause of iron-deficiency anemia in endurance athletes, resulting from repetitive mechanical destruction of erythrocytes during prolonged running. While often subclinical, extreme training volumes may overwhelm compensatory mechanisms and lead to clinically significant anemia that is frequently misattributed to nutritional deficiency or gastrointestinal blood loss. We present a 46-year-old female with mild intellectual disability secondary to traumatic brain injury (TBI) who presented with progressive fatigue and left hip pain. Imaging demonstrated a left femoral neck stress fracture, and laboratory evaluation revealed severe iron-deficiency anemia with a hemoglobin of 8.2 g/dL, elevated total iron-binding capacity, and reduced serum iron, representing a marked decline from pre-injury baseline values. Objective activity monitoring revealed previously unrecognized compulsive endurance exercise behavior, with the patient running 27-32 miles daily for approximately 700 consecutive days. Based on the available clinical data and the patient’s presentation, foot-strike hemolysis-associated iron-deficiency anemia was considered the most likely diagnosis. Temporary cessation of running and mechanical off-loading resulted in complete normalization of hematologic indices within eight weeks, further supporting the presumptive diagnosis. This case highlights the importance of considering mechanical causes of anemia in endurance athletes and demonstrates the value of objective activity assessment, particularly in patients with impaired insight, to prevent delayed diagnosis and unnecessary investigations and interventions.

## Introduction

Historically described as march hemoglobinuria in military recruits, foot-strike hemolysis is now recognized across modern endurance sports, yet remains an underappreciated differential diagnosis of anemia, especially in long-distance runners [1]. This condition is frequently misattributed solely to simple physiologic processes, dietary deficiency, or gastrointestinal blood loss, thus delaying recognition of the mechanical contribution to red cell destruction [1]. In rare cases, severe hemoglobinuria from repetitive mechanical hemolysis has been associated with acute kidney injury, emphasizing the importance of early identification of the underlying stressor [2].

Long-distance running imposes unique hematologic stresses that can lead to measurable red blood cell turnover and, in some athletes, clinically significant anemia [3]. Among these adaptations, exercise-induced hemolysis, described as the destruction of erythrocytes during or shortly after endurance activity, is well documented and arises from a combination of mechanical, metabolic, and oxidative factors inherent to prolonged running [3]. In distance runners, a substantial component of this process is attributed to foot-strike (heel-strike) hemolysis, in which repetitive impact on hard surfaces mechanically fragments erythrocytes within the dense capillary network of the plantar tissues [4]. The cumulative effect of repetitive foot strikes across training sessions may contribute to intravascular hemolysis, particularly in susceptible individuals. This process can overwhelm the reticuloendothelial system, resulting in hemoglobinuria, reduced haptoglobin, and progressive anemia indicating red blood cell destruction [4,5]. However, clinically significant anemia due solely to foot-strike hemolysis appears to be uncommon and requires additional contributing factors.

Although hemolysis in runners is often subclinical, mounting evidence indicates that chronic foot-strike hemolysis may contribute to microcytic anemia, particularly when hemolysis-induced iron loss coexists with the high iron demands of endurance training. Literature supports that repeated hemoglobinuria leads to urinary iron loss, and ongoing red cell destruction increases marrow iron consumption; over time, these mechanisms may deplete iron stores sufficiently to cause microcytosis, low ferritin, and reduced hemoglobin levels [6]. Since these findings can be transient, diagnosis often requires a strong correlation with clinical history and the exclusion of other more common causes of iron-deficiency anemia.

In this case, we describe a 46-year-old female who initially presented to her sports medicine physician with the complaint of hip pain and was subsequently found to have iron-deficiency anemia attributed to presumptive foot-strike hemolysis. Upon further work-up, it was elucidated that her past medical history was significant for a traumatic brain injury (TBI) that had caused her to become unaware of the full extent of her compulsive running, which delayed the recognition of the excessive mechanical loading that was contributing to her presentation. Analysis of a wearable activity monitor provided an objective measure of her exercise volume. This report highlights the importance of including an exhaustive differential diagnosis in the setting of anemia and obtaining a detailed history and considering objectivity monitoring devices, especially in those who have suffered a prior perception-altering event.

## Case presentation

A 46-year-old female with mild intellectual disability secondary to TBI presented to a sports medicine clinic for evaluation of worsening left hip pain. She reported that this pain gradually developed over the last several weeks and was exacerbated by weight-bearing activities, including running, which she reported doing daily. She additionally complained of increasing fatigue. 

Her medical history included TBI, hypercholesterolemia, hypothyroidism, and a chronic seizure disorder. Her home medications were lamotrigine 100 mg three times daily, levetiracetam 1000 mg twice daily, omeprazole 40 mg four times daily, and ferrous sulfate 325 mg three times daily. Surgical history consisted of a left shoulder arthroplasty and a partial colectomy. She had been hospitalized previously following her TBI, which occurred after a fall resulting in stroke and seizure activity; her most recent hospitalization was two years prior. Family history was significant for a stroke in her maternal grandmother. She had no history of alcohol, tobacco, or illicit drug use.

Due to the ongoing and worsening left hip pain, imaging was ordered with initial radiographs not elucidating any remarkable findings. Although the initial radiographs of the hip were normal, MRI revealed a stress fracture of the left femoral neck, specifically a compression side stress reaction that did not need fixation. Upon comprehensive evaluation to establish this patient in the primary care practice, baseline laboratory studies were obtained, including CBC as well as iron studies. Initial laboratory results revealed significant iron deficiency anemia as well as hematuria; these results were compared to iron studies prior to TBI (Table 1).

**Table 1:** Initial laboratory test results

Test	Patient’s Lab Values Prior to Traumatic Brain Injury	Patient’s Initial Visit Lab Values	Reference Ranges
Hemoglobin	12.5 g/dL	8.2 g/dL	11.4-15.2 g/dL
Hematocrit	37.3 %	25.6 %	34-46%
Mean Corpuscular Volume	97 fL	82 fL	80-97 fL
Total Iron Binding Capacity	Not available	498 ug/dL	250-499 ug/dL
Unsaturated Iron-Binding Capacity	Not available	448 ug/dL	155-355 ug/dL
Total Iron	Not available	50 ug/dL	50-212 ug/dL
Hematuria	Not available	Present	Negative

After several further visits, a decision was made by the treating physician to ask the patient if she would be willing to wear a distance tracker on the laces of her running shoes, to which she agreed. Upon review of the running data, it was observed that the patient had been running between 27 and 32 miles. Based on these findings and the patient’s reported extraneous training pattern, it was estimated that the level of activity has been maintained for approximately 700 consecutive days. Given the patient’s running mileage, the laboratory findings consistent with anemia, and the clinical presentation, heel-strike anemia was considered the leading presumptive diagnosis. Because of the concomitant femoral neck stress fracture, the patient was recommended to wear a walking boot for eight weeks. Mechanical off-loading was also intended to assess whether alleviating this repetitive running-related stress would result in improvement of her anemia, thereby providing additional support for the leading diagnosis. Upon returning to the clinic at that time, laboratory tests were recollected and showed complete resolution of her iron deficiency anemia as well as hematuria (Table 2).

**Table 2:** Repeat laboratory test results

Test	Patient’s Lab Values 8 Weeks Post Initial Visit	Reference Ranges
Hemoglobin	12.3 g/dL	11.4-15.2 g/dL
Hematocrit	37.2%	34-46%
Mean Corpuscular Volume	88 fL	80-97 fL
Total Iron Binding Capacity	392 ug/dL	250-499 ug/dL
Unsaturated Iron-Binding Capacity	300 ug/dL	155-355 ug/dL
Total Iron	92 ug/dL	50-212 ug/dL
Hematuria	Negative	Negative

Collectively, as seen in the overview of the case timeline in Figure 1, the patient’s course demonstrated a relationship between TBI and a strenuous running pattern leading to foot-strike hemolysis.

**Figure 1:**
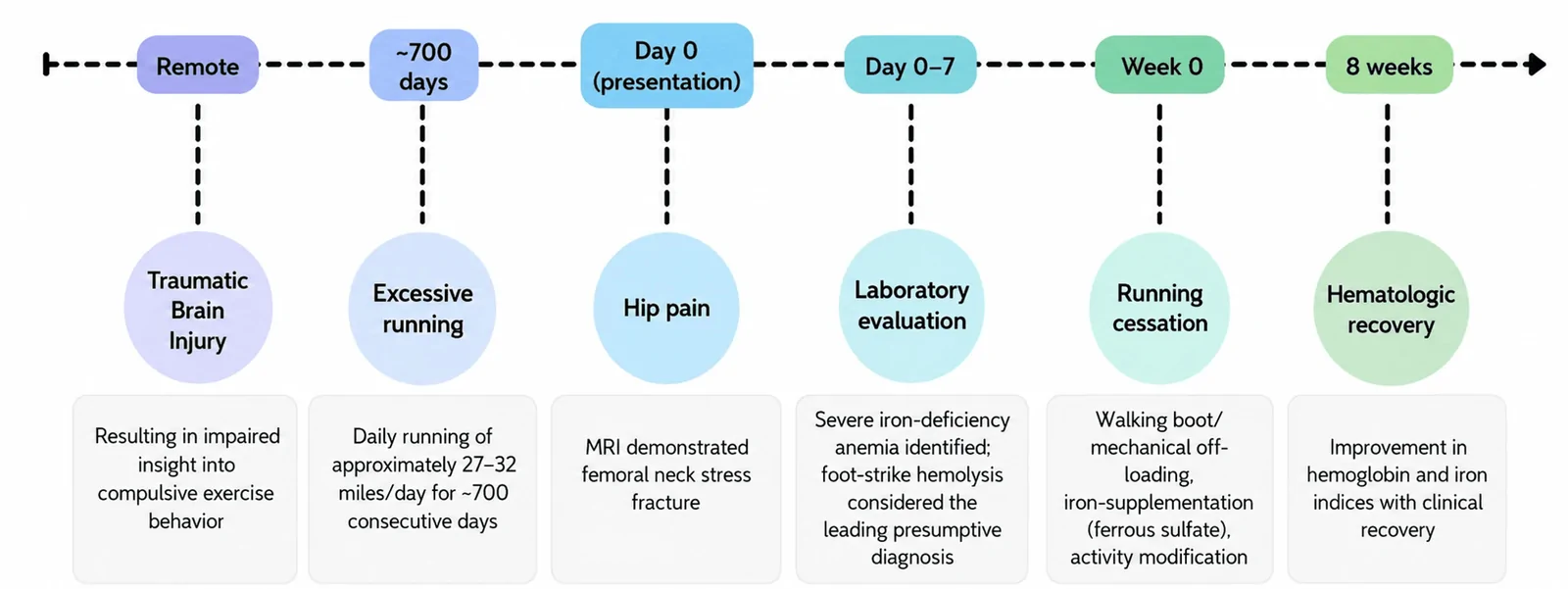
Patient clinical course timeline

## Discussion

This case underscores a rare but important etiology of anemia among endurance runners in foot-strike hemolysis. Foot-strike hemolysis, also referred to as heel strike anemia, may occur as a result of repetitive mechanical trauma during running that leads to intravascular red cell destruction and subsequent iron deficiency. Although described historically as “march hemoglobinuria,” it remains underrecognized as a cause of anemia, particularly when more common explanations such as dietary deficiency or gastrointestinal blood loss are presumed [3]. The predominant mechanism involves mechanical fragmentation of erythrocytes within the capillary networks of the feet and lower limbs during repetitive impact [4,7]. This process releases plasma-free hemoglobin, reduces haptoglobin, and can result in transient hemoglobinuria [7]. In most runners, these effects are mild and compensated; however, in those logging extreme mileage, as in this patient, hemolysis may exceed marrow-replacing capacity, culminating in symptomatic iron-deficiency anemia [5,7].

Anemia in endurance athletes is often multifactorial, involving plasma volume expansion, gastrointestinal micro-bleeding, sweat iron loss, and exercise-induced hepcidin elevation, all of which impair iron balance [8]. In this patient, additional contributors to iron deficiency were also considered, including chronic high-dose omeprazole use and the history of a partial colectomy. Proton pump inhibitor therapy can affect iron absorption due to suppression of gastric acid. Additionally, prior bowel surgery may affect nutrient uptake depending on the extent of the resection as well as the location. Notably, gastrointestinal blood loss is an important consideration in the evaluation of iron-deficiency anemia; however, this patient had no reported symptoms or clinical features suggestive of occult bleeding; therefore, endoscopic evaluation was not pursued during the clinical course. Although these factors may have increased her susceptibility to iron deficiency, the observed laboratory pattern, low serum iron with elevated total iron binding capacity and microcytosis, confirmed true iron deficiency rather than simply a dilutional anemia. Her recovery following temporary cessation of running and use of a walking boot further supports a mechanical rather than nutritional etiology. Although her ferrous sulfate supplement may have contributed to her hematologic recovery, the iron regimen remained unchanged throughout her period of mechanical offloading, making mechanical stress the most likely clinical change associated with the improvement. This discovery of daily running volumes exceeding 30 miles for nearly two years demonstrates the remarkable mechanical stress underlying this presentation. Such repetitive trauma has been associated with iron loss through both hemolysis and urinary excretion of hemoglobin, occasionally leading to renal injury in extreme cases [9]. Moreover, the coexistence of a femoral neck stress fracture was likely multifactorial, with risk factors including the extreme repetitive mechanical loading and possible systemic effects of iron deficiency on bone metabolism and healing [9]. Additionally, other contributors to stress fracture risk and iron deficiency anemia may include relative energy deficiency states, menstrual status, metabolic bone disease, disordered eating behaviors, and thyroid dysfunction. These conditions may independently or in combination with other contributing factors impair a patient’s bone health and/or hematologic status. Although these factors were not directly evaluated in this case, they still remain important considerations when assessing athletes with possible iron-deficiency anemia along with stress fractures. An important consideration in the evaluation of patients with suspected heel-strike anemia is urinalysis, which may provide supportive evidence through the detection of hemoglobinuria resulting from intravascular hemolysis. In this case, the initial urinalysis indicated hematuria, which subsequently resolved after eight weeks, further supporting the leading diagnosis. 

Diagnosis of foot-strike anemia requires a high index of suspicion, especially in patients with unexplained anemia and high endurance training volumes. Objective assessment of activity, such as wearable trackers, can clarify the contribution of excessive mechanical loading when history is limited. Wearable devices may be particularly valuable in patients with cognitive impairment, including patients after TBIs, patients with memory deficits, and those with reduced awareness of behavior changes, due to impaired insight and/or recall resulting in inaccurate self-reported activity levels. This approach provides an objective measure of exercise volume and can reveal patterns of repetitive mechanical loading that otherwise may go unrecognized. In this case, activity monitoring was pivotal to diagnosis and management. Treatment consists primarily of modifying mechanical stressors, reducing mileage, altering running surfaces, or incorporating cross-training, all in combination with iron repletion if indicated [10].

## Conclusions

In summary, foot-strike hemolysis should be considered as a potential contributor to iron deficiency anemia, especially in highly active individuals presenting with otherwise unexplained anemia. Since mechanical destruction of red blood cells can be easily overlooked, it is important to obtain a clear and detailed history, including activity level, especially when symptoms are not supported by clinical findings. More specifically, information regarding daily routine, mileage, and repetitive impact activities should be obtained, as it may reveal stressors that were not initially immediately evident. Incorporating these factors into one’s clinical workup may facilitate earlier recognition of heel-strike anemia, allowing timely correction of anemia and thereby reducing the risk of complications related to anemia and overuse injuries. Early intervention primarily modifies activity levels along with iron repletion if clinically indicated in order to restore hematologic balance. Overall, this case emphasizes the importance of a comprehensive evaluation in athletes with unexplained fatigue or low Hb levels.
